# Moderating Role of State and Trait Positive Affect in Virtual Reality Exposure for Public Speaking Anxiety: Protocol for a Multisite Experimental Study

**DOI:** 10.2196/80010

**Published:** 2025-10-30

**Authors:** Michael W Otto, Laura J Long, Santiago Papini, Tara Culver, Charles T Taylor, Mikael Rubin, Hayley E Fitzgerald, Qimin Liu, Jasper A J Smits, Rebecca A Anderson, Asimina Aslanidou, Christoph Benke, Wouter R Cox, Iris M Engelhard, Sydney E Friedman, Rachel R Goodman, Bronwyn M Graham, Bridget A Hearon, Houston W Howard, Jolene Jacquart, Hannah M Johnson, M Alexandra Kredlow, Charlene L M Lam, Eric B Lee, Grace Vogelzang McClure, Peter M McEvoy, Christiane A Melzig, Tara M Moore, Karen Moses, Samantha M Nagy, Jill M Newby, Paul F R Pfaff, Amy Regan, Winfried Rief, Daniel Rudaizky, Logan H Smith, Sharon C Sung, Matthias J Wieser, Alex H K Wong, Quincy J J Wong, Nur Hani Zainal, Zhiqi Zhang

**Affiliations:** 1 Department of Psychological and Brain Sciences, Boston University, 64 Cummington Mall, Boston, MA, 02215, United States, 1 617-353-9610; 2 Exposure Therapy Consortium, The University of Texas, Austin, United States; 3 Department of Psychology, University of Hawaiʻi at Mānoa, Honolulu, HI, United States; 4 Department of Psychiatry, University of California, San Diego, La Jolla, CA, United States; 5 Department of Psychology, Palo Alto University, Palo Alto, CA, United States; 6 Department of Psychology, The University of Texas at Austin, Austin, TX, United States; 7 enAble Institute and School of Population Health, Curtin University, Bentley, Australia; 8 Department of Psychology, Educational Science, and Child Studies, Erasmus University Rotterdam, Rotterdam, The Netherlands; 9 Department of Clinical Psychology, Experimental Psychopathology and Psychotherapy, Philipps University of Marburg, Marburg, Germany; 10 Department of Clinical Psychology, Utrecht University, Utrecht, The Netherlands; 11 Department of Psychology, University of Arizona, Tucson, AZ, United States; 12 Department of Psychology, Tufts University, Medford, MA, United States; 13 School of Psychology, UNSW Sydney, Sydney, Australia; 14 Department of Psychology, Albright College, Reading, PA, United States; 15 School of Psychological and Behavioral Sciences, Southern Illinois University Carbondale, Carbondale, IL, United States; 16 Department of Psychology, Harvard University, Cambridge, MA, USA; 17 Laboratory of Clinical Psychology and Affective Neuroscience, University of Hong Kong, Hong Kong, China (Hong Kong); 18 State Key Laboratory of Brain and Cognitive Sciences, The University of Hong Kong, Hong Kong, Hong Kong Special Administrative Region of China; 19 Centre for Clinical Interventions, Northbridge, Australia; 20 School of Psychology, Western Sydney University, New South Wales, Australia; 21 Black Dog Institute, Sydney, Australia; 22 Department of Clinical Psychology and Psychotherapy, Philipps University of Marburg, Marburg, Germany; 23 Programme in Health Services and Systems Research, Duke-NUS Medical School, Singapore, Singapore; 24 Department of Psychology, National University of Singapore, Singapore, Singapore

**Keywords:** team science, exposure therapy, moderators, state affect, trait positive affect, speech anxiety, virtual reality

## Abstract

**Background:**

The identification of moderators of exposure-based cognitive behavioral therapy (EXCBT) outcomes has the potential to (1) guide the judicious application of the limited resource that is EXCBT and (2) identify additional treatment targets for ameliorating the deleterious effects of an identified moderator, thereby enhancing clinical response. Experimental and clinical studies have yielded intriguing findings for the moderating influence of positive affect on EXCBT outcomes. Mixed findings for state positive affect (at the time of extinction or exposure trials) as a predictor of EXCBT outcomes stand in contrast to evidence that baseline levels of trait positive affect may be a more effective predictor. As such, questions remain about the best way to assess positive affect as a potential treatment moderator.

**Objective:**

This study was designed to investigate (1) the relative value of state and trait positive affect for predicting the outcome of a single-session virtual reality exposure intervention for public speaking anxiety in adults attending college and (2) the role of 3 related constructs—optimism, hopefulness, and mental health self-efficacy—that may explain the predictive significance of trait positive affect.

**Methods:**

State affect will be manipulated at an experimental level using affect induction procedures; trait positive affect will be measured at baseline. Three dependent measures—a primary outcome of public speaking anxiety and secondary outcomes of social phobia and self-reported valence—will be examined. This study relies on a team science approach and is being conducted across 12 collaborating sites through the Exposure Therapy Consortium, allowing for replication of all findings across diverse study sites worldwide.

**Results:**

Data collection for this study began in October 2024 and ended in August 2025. We expect to complete data analysis and submit results for publication in approximately November 2025.

**Conclusions:**

This study will help clarify the relationship among positive affect (state and trait), exposure learning, and 3 related constructs (optimism, hopefulness, and mental health self-efficacy). These findings will illuminate strategies for future treatment improvement and enhanced efficacy.

## Introduction

### Background

Exposure-based cognitive behavioral therapy (EXCBT) is an efficacious first-line treatment for anxiety-related disorders (ie, anxiety, obsessive-compulsive, and trauma- and stressor-related disorders) [1-6]. However, despite its clear efficacy, a substantial minority of patients fail to respond adequately to EXCBT [7-9], continuing the distress and loss of productivity associated with these disorders [10-12]. These limitations on efficacy have motivated the study of extinction learning processes and potential moderators of these processes to better understand, strengthen, and target EXCBT efficacy [7,9].

One such moderator of interest is the affective context of the exposure. In a comprehensive review of the potential of positive affect for improving EXCBT outcomes, Zbozinek and Craske [13] considered the potential beneficial effects of positive affect on core processes linked to extinction learning and retention: attention, encoding, rehearsal, consolidation, retrieval, and stimulus valence. Empirical tests of these ideas have been ongoing. Initial significant findings have been observed for experimental paradigms where positive versus neutral affect were induced [14] or naturally occurring positive affect was examined [15] for predicting better fear conditioning outcomes. However, beneficial effects for positive versus negative affect induction before extinction learning trials were not replicated by Fitzgerald [16] using a fear conditioning paradigm or by van Veen et al [17] using 2-session virtual reality (VR) exposure for participants with high public speaking anxiety. Failures to observe a beneficial effect for preexposure positive affect have also been reported in studies examining extant levels of positive affect in individuals undergoing treatment [18,19].

One possible explanation for these inconsistent findings is that positive affect needs to be experienced across multiple phases of exposure learning, that is, before, during, and after exposure, when the therapeutic memory is consolidated or reconsolidated (eg, when reflecting on the exposure or during between-session exposure practices) [20]. If this explanation is accurate, trait positive affectivity should be a better predictor than state positive affectivity because it has the opportunity to exert its hypothesized effects at multiple phases of the exposure learning process. Results of predictor analyses from 2 clinical trials support this hypothesis [21]. These studies by Taylor et al [22,23] used a measure of trait positive emotionality drawn from the extraversion domain of the NEO Five-Factor Inventory (NEO-FFI) [24], and both studies predicted a significantly better response to cognitive behavioral therapy.

However, trait positive affect is arguably a broader assessment domain than state affect, as reflected by the nature of the assessments used by Taylor et al [22,23]. Specifically, in their first study, Taylor et al [22] used an 8-item positive emotion scale from the extraversion domain of the NEO-FFI, with items reflecting levels of happiness, joy, excitement, and optimism characteristic of one’s personality. The second trial [23] used a shortened assessment: a 4-item positive emotion scale again derived from the extraversion domain of the NEO-FFI. Although Taylor et al [22,23] controlled for associations with the broader domain of extraversion and negative mood reflecting depression, potential elements or correlates of trait positive emotionality may help explain the ability of positive emotionality to predict treatment outcomes. For example, there are a number of variables that are correlated with both positive affect and treatment responsivity and outcomes and, hence, may account for the predictive significance of trait positive affect. Specifically, 3 additional variables—optimism, hopefulness, and self-efficacy—emerge as important assessment targets. Each was selected due to its potential to mediate effects of trait positive affectivity on EXCBT, as indicated by significant associations between each of these variables and positive affect in addition to significant associations with adaptive responses to stress, adaptive coping, positive expectancies, and positive treatment outcomes [25-35]. Accordingly, to hone prediction and better understand the nature of trait positive affect as a potential moderator of EXCBT outcomes, it is important to evaluate whether the prediction hypothesized for trait positive affect can be accounted for by related constructs such as state positive affect, optimism, hopefulness, and self-efficacy.

### Study Objectives

This preregistered study is designed to enhance the understanding of positive affect as a potential predictor (moderator) of EXCBT outcomes by examining two related lines of inquiry: (1) Can we confirm that trait positive affect is a better predictor of treatment outcomes than state positive affect? (2) Can the prediction based on trait positive affect be better understood by considering 3 alternative assessment targets: optimism, hopefulness, and self-efficacy? Answering these questions has the potential to hone attention to a correct and specific mechanistic target (trait vs state positive affect) for understanding and prospectively intervening, with individual characteristics predicting nonresponse to EXCBT. Specifically, accurate specification of a marker of relative nonresponse to this treatment can have a public health impact by aiding (1) the judicious application of EXCBT (personalized medicine) and (2) pretreatment efforts to modify the moderator. Indeed, there is preliminary evidence that such affect-focused treatments can be successful [36]. Accordingly, the longer-term potential of this study is to provide additional strategies for the effective use of EXCBT for patients in need.

To assess the relative capacity of state and trait positive affect to predict responsivity to exposure therapy, we will examine both constructs as moderators of the degree of improvement from a single-session virtual reality exposure intervention for public speaking anxiety in college students. We selected public speaking anxiety as the therapeutic target because it is a prevalent concern among undergraduate students [37,38] and because the use of VR exposure has demonstrated efficacy for treating public speaking anxiety and other social anxiety symptoms [17,39,40]. Furthermore, a VR-delivered intervention is also a highly standardized procedure that can be reproduced exactly across participants and sites [40]. Finally, the use of a single-session VR exposure intervention to judge extinction learning approximates the intervention procedures used by van Veen et al [17] in their study of positive affect induction and exposure outcomes, as well as laboratory paradigms [14].

In this study, state affect just before exposure will be manipulated at an experimental level using affect induction procedures. Trait positive affect will be measured at baseline using procedures developed by Taylor et al [23]. Randomized affect induction will be used for two reasons: (1) to allow for comparison to previous studies that used similar methods [14,16,17] and (2) to ensure that state affect at the time of extinction will be distinct from the degree of trait positive affect. In addition, some studies that have found benefits of state positive affect for extinction learning and return-of-fear outcomes have used the Positive and Negative Affect Schedule [14,41] and, thus, may have been influenced by arousal given that the Positive and Negative Affect Schedule includes only high-arousal items [15]. Our inclusion of state arousal based on the Self-Assessment Manikin (see the Methods section) will help address this alternative explanation for conflicting state affect findings and the value of considering trait affect over state measures. Three dependent measures will be examined—a primary outcome of public speaking anxiety and secondary outcomes of social anxiety and self-reported valence to giving a speech—measured at the 1-week follow-up as the primary outcome window. Initial analyses will evaluate the capacity of both state and trait positive affect to predict VR exposure outcomes. Subsequent analyses will evaluate the degree to which the predictive value of trait positive affect can be accounted for or improved by considering measures of optimism, hopefulness, and self-efficacy.

### Use of a Team Science Approach

Replication issues in psychological science have been well documented [42,43], motivating efforts to address them at the study design stage, including efforts to increase both the size and representativeness of the samples under study [44,45]. One strategy for achieving this goal is to conduct team science, defined as a “method involving a relatively large number of collaborators who may be dispersed across labs, institutions, disciplines, cultures, and continents” [46]. The goal is to facilitate replicable research by helping researchers pool their resources to reduce study burden while increasing the size and representativeness of the study sample.

In accordance with this recommendation, this study was designed and proposed and will be conducted by members of the Exposure Therapy Consortium (ETC [47] ) [9]. This study responds to recommendations for team science [46] by using a consensus design (ie, examined and approved by the ETC Rigor and Reproducibility Committee and the research steering committee [9]); multiple measures for select constructs; strong documentation of materials and methods; a large and diverse sample; collaboration with colleagues from different settings, backgrounds, and cultures; registration of the trial and analytic plan; and a plan to share data and analytic codes through Open Science Framework registration. To this end, this team science approach will not only enhance the replicability of this study but also enable a more comprehensive investigation of the intricate interplay between positive affect and exposure therapy outcomes across diverse populations and settings.

## Methods

### Sites

This is a multicenter study with 12 participating sites. Boston University serves as the lead site (coordinating center), distributing study methods and materials to the collaborating investigators at Albright College, Curtin University, Erasmus University Rotterdam, Southern Illinois University, Tufts University, University of Arizona, University of Hong Kong, Marburg University, University of New South Wales, University of Texas at Austin, and Utrecht University. Adherence to study procedures is aided by videotaped procedures distributed to the sites. All data entry is conducted via self-report using a REDCap (Research Electronic Data Capture; Vanderbilt University) interface. Each site is asked to screen up to 150 participants, from whom up to 80 participants (the minimal target sample size per site is 40) are to be selected for the VR exposure intervention and posttreatment and follow-up assessments. If needed, each site is permitted to recruit beyond 150 participants for the screening phase to reach the target sample size in the intervention phase. This study is registered with ClinicalTrials.gov (NCT06593847).

### Participants

Participants for this study will be undergraduate students reporting high levels of public speaking anxiety who are willing to engage in a single-session intervention. Inclusion criteria for the screening phase of this study (see the Procedures section) are self-reported age of between 18 and 70 years, current attendance to college, ability to read the dominant language used at the university, familiarity with a keyboard and mouse or touch screen device, and willingness to provide informed consent through the REDCap interface.

### Assessments

#### Overview

Table 1 indicates the study phase in which each variable is assessed. Demographic information will be obtained using a brief questionnaire at screening, and an additional treatment history form will be used to obtain clinical information regarding mental health diagnoses and previous mental health treatment; this information will be used to characterize the sample. All outcomes are self-reported via REDCap.

**Table 1.** Assessment schedule.

Measure	Screening and baseline assessment	In-person VREX^a^ visit	1-week follow-up assessment
Selection and outcome variables			
Demographics	✓		
PRCA-PA^b^	✓	✓	✓
Valence	✓	✓	✓
SPIN^c^	✓		✓
Core predictor variables			
SAM^d^		✓	
Trait positive affect	✓		
AHS^e^	✓		
LOT-R^f^	✓		
MHSES^g^	✓		
Additional measures			
RRQ^h^	✓		
SCI^i^	✓		
CAMS-R^j^	✓		
SBI^k^	✓		
Treatment history	✓		

a VREX: virtual reality exposure.

b PRCA-PS: Personal Report of Communication Apprehension, Public Speaking Subscale.

c SPIN: Social Phobia Inventory.

d SAM: Self-Assessment Manikin.

e AHS: Adult Hope Scale.

f LOT-R: Life Orientation Test–Revised.

g MHSES: Mental Health Self-Efficacy Scale.

h RRQ: Ruminative Response Questionnaire.

i SCI: Sleep Condition Indicator.

j CAMS-R: Cognitive and Affective Mindfulness Scale–Revised.

k SBI: Savoring Beliefs Inventory.

#### Selection and Outcome Variables

The Personal Report of Communication Apprehension, Public Speaking Subscale (PRCA-PS), is a 6-item self-report measure for assessing level of public speaking anxiety; item responses range from 1 (“strongly disagree”) to 5 (“strongly agree”), providing total scores that can range from 6 to 30 [48]. Scores of 18 or higher indicate high- to moderate-severity public speaking anxiety and are used to select individuals for this study at the screening and baseline assessment. In a pilot study for this trial [49], 81.3% of the screened sample responding to advertisements for a public speaking anxiety study scored 18 or higher on the PRCA-PS, consistent with high rates of social anxiety reported for student populations [37,38]. The PRCA-PS serves as the primary outcome at the 1-week follow-up assessment.

The Social Phobia Inventory is a 17-item self-report scale to assess fear, avoidance, and physiological symptoms associated with social anxiety disorder over the previous week [50]. The total score (which can range from 0 to 68) will be used as a measure of social anxiety severity, a secondary outcome variable, and will be assessed at the screening and baseline assessment and 1-week follow-up assessments.

The valence of giving speeches is rated on a scale ranging from −3 (extremely unpleasant) to +3 (extremely pleasant). This measure will be administered at the screening and baseline assessment, immediately after treatment, and at the 1-week posttreatment assessment.

#### Core Predictor Variables

The Self-Assessment Manikin uses pictures of a humanlike form to have participants rate the degree of current pleasure, emotional arousal, and dominance by selecting the figure that most represents their current affective state on each of these dimensions [51]. The pleasure rating (ranging from 1 to 9) will be used as the measure of state positive affect, administered before and after the affect induction. In regression equations, prediction will also be examined for degree of state affective arousal.

Trait positive affect will be assessed at the screening and baseline assessment with the 4 items used by Taylor et al [23] drawn from the extraversion domain of the NEO-FFI (laughs easily, cheerful, and vivacious; reverse items: not cheerful or lighthearted and not a cheerful optimist) [24]. The total of these items rated on a 5-point Likert scale (with scores ranging from 0 to 16) will be used as a predictor of VR exposure outcome.

The Adult Hope Scale is a 12-item measure that assesses hope, a positive motivational resource consisting of agency, or goal-directed energy, and the capacity to identify pathways toward goals [52]. The total score (which can range from 8 to 64) will be used as a predictor of VR exposure outcomes.

The Life Orientation Test–Revised is a 6-item (with 4 additional filler items) measure of optimism: the dispositional tendency to expect positive outcomes of events [53]. The total score, which can range from 0 to 24, will be used as a predictor of VR exposure outcome.

The Mental Health Self-Efficacy Scale is a 7-item measure of self-efficacy for engaging in mental health–promoting behaviors, including the ability to accept uncomfortable mental events, emotions, or sensations; refrain from maladaptive coping or protective actions; and engage in health-promoting behaviors and valued activities (Tech, MJ, unpublished data, April 2025). The mean confidence rating for these items (expressed as between 0% and 100% confidence in the ability to engage in these behaviors) will serve as the self-efficacy measure for predicting VR exposure outcomes.

#### Additional Measures

The following measures, used to assess degree of rumination, sleep disruption, mindfulness, and savoring, will be assessed at baseline and used in independent, exploratory analyses of associations with VR exposure treatment outcomes given findings indicating such associations [54-57]: the Ruminative Response Questionnaire, a 22-item measure assessing an individual’s tendency to ruminate when feeling sad, down, or depressed [58]; the Sleep Condition Indicator (SCI), an 8-item measure designed to assess an individual’s sleep quality over the previous month [59]; the Cognitive and Affective Mindfulness Scale–Revised, a 10-item unidimensional scale that assesses mindfulness as it is experienced in general daily occurrences [60]; and the Savoring Beliefs Inventory, a 24-item scale that assesses the tendency to savor, or amplify, positive emotions [61].

### Procedures

Select aspects of study procedures may vary from site to site (eg, follow-up session completed in person vs remotely); however, all core procedures (ie, affect induction, VR exposure, and self-report measures) remain consistent. To promote standardization of procedures across sites, study personnel were trained using an instructional video and followed a scripted protocol when implementing VR exposure; sites were also asked to track any deviations. This clinical trial is registered on both ClinicalTrials.gov (NCT06593847) and the Open Science Framework (4ucmj). No changes to the protocol, interim analyses, specific stopping rules, or external monitoring other than by the institutional review boards (IRBs) or health research ethics committees (HRECs) are planned for this study.

### Ethical Considerations

All collaborating sites received IRB or HREC approval before data collection. IRB or ethics committee approval numbers were as follows: Boston University (7565E) for which Albright College, the University of Texas at Austin, and Southern Illinois University were reliant upon; Curtin University (HRE2025-0016); Erasmus University (ETH2425-0422); Tufts University (STUDY00005259); University of New South Wales (iRECS 7416); University of Hong Kong (EA240580); University of Marburg (2024-81k); University of Arizona (STUDY00004970); and Utrecht University (FETC 25-0150). All sites provided participants with compensation through either course credits, extra credit, and or monetary compensation.

### Screening and Baseline Procedures

Interested participants responding to an advertisement for a “Virtual Reality Intervention for Speech Anxiety Study” will be given access to an online survey via REDCap [62]. Participants will be provided with an electronic consent form explaining all study procedures. If they agree to participate, they will click a button indicating their consent and will be told to continue to the screening and baseline survey. On the basis of responses to the survey (including fully completing core measures, responding correctly to quality assurance items, and completing the survey within 5 days of initiating it), eligible participants meeting the PRCA-PS selection score (>18) will be invited to the in-person session.

### In-Person Study Session

The in-person study session lasts approximately 75 minutes and begins with an orientation to the VR device. Participants will put on the headset while sitting in a chair. Participants will be oriented to the VR environment by briefly viewing a 360-degree nature scene. Following VR orientation, participants will be asked to rate their current affect and will then watch 1 of 2 short film clips (totaling approximately 5 minutes) for either positive or negative affect induction, determined via random block assignment (1:1 allocation) using an online tool [63], with a block size of 6 and separate blocks for individuals whose sex assigned at birth was male versus female, as programmed into REDCap. Participants are blind to hypotheses. Film clips are shown on a separate laptop or tablet screen. Film clips for positive affect induction were sourced from the movie *Wall-E* and from the television show *Whose Line Is It Anyway?* The film clips for negative affect induction were sourced from the movies *Bambi* and *The Champ*. Studies have demonstrated that each of these film clips is effective in eliciting the targeted affect [64-67]. Before viewing the film clips, participants will be provided with the following instructions: “Let yourself experience whatever emotions you have, as fully as you can. Don’t try to hold back or hold in your feelings.” Participants rerate their state affect following their randomized affect induction procedures.

Following affect induction and subsequent state affect ratings, participants will be shown a prerecorded exposure therapy instruction provided by a therapist from within the VR headset. Participants undergo 6 exposure trials wherein they give a speech to a virtual audience of 6 individuals sitting in chairs around a conference table or in a lecture hall in the 360-degree VR environments [68]. The topic for the speech is “something you’re proud of.” Following exposures, the VR therapist congratulates the participant and provides a short debriefing statement. Once they remove the headset, participants will be asked to rate their public speaking anxiety and current valence toward giving a speech.

### Follow-Up Outcome Assessment

One week following the completion of the single-session VR exposure intervention, participants will be scheduled for the 1-week follow-up assessment. This survey is expected to take approximately 15 minutes to complete. Participants will receive course credit for the portion of the study in which they participate. Spontaneously reported adverse events will be reported to site IRBs or HRECs.

### Data Sharing

Data will be analyzed and published by participating ETC investigators. Following publication of primary papers, deidentified data will be available to researchers who sign a data use agreement.

### Data Analysis

The preregistered analysis plan on the Open Science Framework provides specifics on the tests to be conducted. In general, a linear mixed modeling approach will be used for data analysis, examining the overall and unique prediction afforded by predictor variables of interest while accounting for variance across sites. Preliminary analyses will evaluate the effects of the experimental manipulation (affect induction) on state affect. Preliminary analyses will also examine the relationships among predictors, particularly in relation to the degree to which trait positive affect shares variance with the hypothesized related constructs of interest: optimism, hopefulness, and self-efficacy. Core analyses will use linear mixed models to examine the hypothesis that trait positive affect is a stronger predictor of outcomes than state positive affect (also accounting for state affective arousal). Core tests of hypotheses will also examine the shared and unique prediction of outcomes, relative to trait positive affect, of measures of optimism, hopefulness, and self-efficacy. The goal of these analyses is to identify the strongest predictors of outcomes to provide greater clarity on the role of positive affect (and associated constructs) as a potential moderator of outcome of exposure-based interventions.

To address missing data and dropouts, we will implement a multipronged approach. The primary analysis will follow intention-to-treat principles using multiple imputation by chained equations, incorporating all available baseline predictors, affect condition assignment, and site indicators in the imputation model. For sensitivity analyses, we will conduct complete case analysis to compare with imputed results.

Power analyses were conducted using the following assumptions: α of .05, power of 80%, and a mixed-effects modeling approach accounting for clustering within sites. We examined detectable effect sizes across a range of intraclass correlation coefficients (ICCs=0.01 to 0.30) to account for variability in site-level clustering that is commonly observed in multisite studies. With our planned sample sizes (40-80 participants per site across 12 sites), this study is adequately powered to detect clinically meaningful moderation effects under realistic ICC assumptions. For example, with low clustering (ICC≤0.05), this study can detect moderation effects of Cohen *d*=0.36 to 0.48, representing medium effect sizes that are clinically meaningful.

## Results

A pilot study of the efficacy of this VR exposure intervention for public speaking anxiety in a sample of 47 college students (who had undergone positive and negative affect induction procedures before exposure) indicated strong feasibility for the methods of this study. The results showed beneficial effects of the single-session VR exposure intervention as indicated by a small to moderate effect size for public speaking anxiety (*d*=0.32), a moderate effect size for social phobia symptoms (*d*=0.54), and a small to moderate effect size for self-reported speech valence (*d*=0.44) from baseline to the 1-week follow-up [49]. Data collection for this study began in October 2024 and is projected to ended in August 2025. We expect to complete data analysis and submit results for publication in approximately October 2025.

## Discussion

The ETC was organized to *generate better data faster* by facilitating larger-scale studies that can be completed without substantial funding due to relatively low burden across multiple collaborating sites [9,47]. Furthermore, the use of a particularly large and diverse sample recruited at study sites reflecting different settings, backgrounds, and cultures addresses both issues of lower power and limited replication that have characterized the study of the influence of state and trait positive affect on extinction or EXCBT outcomes [13-19,21-23]. The findings being achieved across the various sites in this trial will provide a perspective on their robustness. In addition, although power to detect specific site differences is limited across a 12-site study, discussions of results will be summarized in relation to an ICC used to describe the amount of variability in prediction that can be attributed to cross-site differences.

A limitation of this study is the focus on the potentially homogeneous population of undergraduate students with high public speaking anxiety; the degree to which our results will fully generalize to a clinical population is not known. Accordingly, although this trial will provide a well-honed test of the role of positive affectivity in response to a well-defined exposure therapy intervention, replication of these methods will be needed for clinical samples. This study also relies on self-report instruments without the use of performance or physiological outcomes. This study is designed to address the influence of positive affectivity on acute (1 week) exposure outcomes; the influence of exposure on the maintenance of treatment gains awaits future study.

The accurate identification of markers of EXCBT’s acute efficacy is important given that EXCBT is a limited resource, with evidence that only a minority of individuals receive EXCBT for anxiety-related disorders despite its status as a first-line treatment 69-71]. This study was designed to provide guidance on the role of positive affect in treatment response to aid personalized medicine decisions, including, perhaps, pretreatment efforts to modify the moderator [36,67,68].

## Supplementary material

10.2196/80010 Checklist 1 SPIRIT checklist.

## Abbreviations

ETC: Exposure Therapy Consortium
EXCBT: exposure-based cognitive behavioral therapy
HREC: health research ethics committee
ICC: intraclass correlation coefficient
IRB: institutional review board
NEO-FFI: NEO Five-Factor Inventory
PRCA-PS: Personal Report of Communication Apprehension, Public Speaking Subscale
REDCap: Research Electronic Data Capture
SPIRIT: Standard Protocol Items: Recommendations for Interventional Trials
VR: virtual reality
